# Suppression of Mouse AApoAII Amyloidosis Progression by Daily Supplementation with Oxidative Stress Inhibitors

**DOI:** 10.1155/2019/1263274

**Published:** 2019-06-04

**Authors:** Jian Dai, Xin Ding, Hiroki Miyahara, Zhe Xu, Xiaoran Cui, Yuichi Igarashi, Jinko Sawashita, Masayuki Mori, Keiichi Higuchi

**Affiliations:** ^1^Department of Aging Biology, Institute of Pathogenesis and Disease Prevention, Shinshu University Graduate School of Medicine, Matsumoto 390-8621, Japan; ^2^Department of Biological Sciences for Intractable Neurological Diseases, Institute for Biomedical Sciences, Interdisciplinary Cluster for Cutting Edge Research, Shinshu University, Matsumoto 390-8621, Japan; ^3^The First Hospital of Hebei Medical University, Shijiazhuang 050030, China; ^4^Supplemental Nutrition Division, Pharma & Supplemental Nutrition Solutions Vehicle, Kaneka Corporation, Osaka 530-8288, Japan; ^5^Department of Advanced Medicine for Health Promotion, Institute for Biomedical Sciences, Interdisciplinary Cluster for Cutting Edge Research, Shinshu University, Matsumoto 390-8621, Japan

## Abstract

Amyloidosis is a group of diseases characterized by protein misfolding and aggregation to form amyloid fibrils and subsequent deposition within various tissues. Previous studies have indicated that amyloidosis is often associated with oxidative stress. However, it is not clear whether oxidative stress is involved in the progression of amyloidosis. We administered the oxidative stress inhibitors tempol and apocynin via drinking water to the R1.P1-*Apoa2^c^* mouse strain induced to develop mouse apolipoprotein A-II (AApoAII) amyloidosis and found that treatment with oxidative stress inhibitors led to reduction in AApoAII amyloidosis progression compared to an untreated group after 12 weeks, especially in the skin, stomach, and liver. There was no effect on ApoA-II plasma levels or expression of *Apoa2* mRNA. Detection of the lipid peroxidation markers 4-hydroxynonenal (4-HNE) and malondialdehyde (MDA) revealed that the antioxidative effects of the treatments were most obvious in the skin, stomach, and liver, which contained higher levels of basal oxidative stress. Moreover, the unfolded protein response was reduced in the liver and was associated with a decrease in oxidative stress and amyloid deposition. These results suggest that antioxidants can suppress the progression of AApoAII amyloid deposition in the improved microenvironment of tissues and that the effect may be related to the levels of oxidative stress in local tissues. This finding provides insights for antioxidative stress treatment strategies for amyloidosis.

## 1. Introduction

Amyloidosis is a group of diseases in which abnormal protein aggregates, known as amyloid fibrils, build up in the brains of patients with Alzheimer's disease (AD), as well as in various organs in cases of amyloid light-chain (AL) amyloidosis, transthyretin (ATTR) amyloidosis, and mouse apolipoprotein A-II (AApoAII) amyloidosis [1–3]. Amyloidosis is a serious health problem that can lead to life-threatening organ failure. Aggregation of amyloid proteins proceeds via structurally unstable amyloid proteins or precursor proteins under certain conditions, such as overproduction of amyloid proteins, mutation, enzymatic cleavage of precursor proteins, low pH, and aging, resulting in a transition to an unstable protein state [2, 4–7]. Recent evidence suggests that oxidative modification of amyloid proteins may also be one of these factors that may lead to an increase in aggregation propensity [8–10]. Amyloid fibril formation is a gradual process that involves conformationally modified monomers, oligomers, and protofibrils. Most of the intermediates have been shown to be cytotoxic and stimulate stress and immune responses in the cells around the areas of deposition [11–13]. Increased levels of oxidative stress around the amyloid deposits have been detected in a variety of amyloid diseases [12–16].

Apolipoprotein A-II (ApoA-II) is the second most abundant protein in high-density lipoprotein (HDL) particles and is involved in lipid metabolism [17]. However, the exact functions of ApoA-II protein remain unclear. Some studies have found that ApoA-II protein is related to lipid transport from peripheral organs to the liver and binding of plasma proteins with HDL [17–19]. In a previous study, we found that ApoA-II modifies the binding of the plasma acute phase inflammatory response protein serum amyloid A (SAA) with plasma lipoproteins and plays a role in regulating the inflammatory response and AA amyloid fibril formation [20]. We found that ApoA-II proteins form amyloid fibrils (AApoAII) and deposit extracellularly in a number of organs (except the brain) and are associated with aging in senescence-accelerated mouse prone-1 (SAMP1) strains of mice. We later identified AApoAII deposits in various strains of mice [3, 21]. We demonstrated that there are seven ApoA-II alleles among various strains of mice. Among them, amyloidogenic C-type ApoA-II protein (APOA2C) was found to form AApoAII amyloid fibrils in mice associated with earlier aging than the other types of ApoA-II [22]. A congenic strain of mice with the amyloidogenic *Apoa2^c^* allele on the genetic background of the senescence-accelerated resistant mouse 1 (SAMR1) strain was developed and named R1.P1-*Apoa2^c^* mice [3]. Our previous research revealed that AApoAII amyloidosis is transmitted in a prion-like manner and is induced by the intake of amyloid fibrils through feces, saliva, breast milk, and blood [23–25]. R1.P1-*Apoa2^c^* mice rarely exhibit spontaneous AApoAII amyloidosis under specific pathogen-free (SPF) conditions, but intravenous injection of a small amount of AApoAII amyloid fibrils induces AApoAII amyloidosis at high reproducibility [3].

In amyloidosis studies, amyloid deposition induced rising oxidative stress in surrounding cells and tissues and led to subsequent cell dysfunction and elevation of apoptosis markers [12–15], which is considered to be a major aspect of organ impairment associated with amyloidosis. There is sufficient experimental evidence from Alzheimer's disease research suggesting that nerve cell degeneration is mainly due to damage caused by oxidative stress, both in animals and in humans [26, 27]. Our previous studies also found that AApoAII amyloid deposition induced both unfolded protein response (UPR) and endoplasmic reticulum (ER) stress in the liver and kidney, leading to an increase of apoptotic cells [28]. On the other hand, studies of localized amyloidosis found that increases in oxidative stress preceded A*β* deposition, which may promote subsequent protein aggregation [29, 30]. In other studies, it was hypothesized that oxidative stress induces protein aggregation [7–9]. It was reported that TTR protein treated with oxynitride exhibited higher aggregation activity *in vitro*, which may be caused by structural instability of the modified amyloid protein [9]. However, satisfactory evidence does not exist to prove the role that oxidative stress plays in the process of amyloid deposition, especially in systemic amyloidosis.

Aging is a strong common feature of amyloidosis, except for early-onset amyloidosis caused by genetic mutations or overproduction of amyloid protein by acute phase immune reaction and cancer [31, 32]. Considering the important role of oxidative stress in various age-related diseases, we hypothesized that age-associated oxidative stress is also involved in the pathogenesis of AApoAII amyloidosis. Our recent study found that following caloric restriction, the degree of amyloid deposition in mice decreased significantly and was accompanied by downregulation of expression of genes associated with both oxidative stress and aging in the liver [33]. It is not clear whether the decline in oxidative stress levels caused the suppression in AApoAII amyloidosis.

Although the importance of oxidative stress during the progression of amyloidosis has been recognized, it needs to be determined whether oxidative stress is the cause or the consequence of amyloid deposition. To address this critical knowledge gap, we examined the effect of the nonspecific reactive oxygen species (ROS) scavenger tempol and the NADPH oxidase inhibitor apocynin on the process of systemic amyloidosis using our unique amyloid analyzing system of mouse AApoAII amyloidosis.

## 2. Materials and Methods

### 2.1. Animals

We used female R1.P1-*Apoa2c* congenic mice, which carry the amyloidogenic type c allele (*Apoa2c*) from the AApoAII amyloidosis-susceptible SAMP1 strain on a genetic background of the SAMR1 strain. R1.P1-*Apoa2^c^* mice exhibit normal aging and do not develop AApoAII amyloidosis under specific pathogen-free (SPF) conditions, whereas they develop accelerated AApoAII amyloidosis by oral or intravenous administration of AApoAII fibrils [3]. R1.P1-*Apoa2^c^* mice were maintained in our laboratory.

Mice were raised under SPF conditions at 24 ± 2°C with a light-controlled regimen (12-hour light/dark cycle, lights on at 9:00 and off at 21:00) in the Division of Animal Research, Research Center for Supports to Advanced Science, Shinshu University. Mice were raised on a commercial diet (MF, Oriental Yeast, Tokyo, Japan) and tap water. Three to 6 female mice housed in a single cage were used for experiments to avoid the anticipated adverse impacts due to fighting among male mice housed in the same cage.

Mice were sacrificed by cardiac puncture under deep sevoflurane anesthesia. For biochemical analysis, plasma and a portion of the organs were snap-frozen by liquid nitrogen and stored at -80°C. For histochemical analysis, the remaining organs were fixed in 10% neutral buffered formalin followed by embedding in paraffin. All experiments were performed with the approval of the Committee for Animal Experiments of Shinshu University (Approval No. 280086).

### 2.2. Experimental Design

#### 2.2.1. Drug Administration

**Figure 1:**
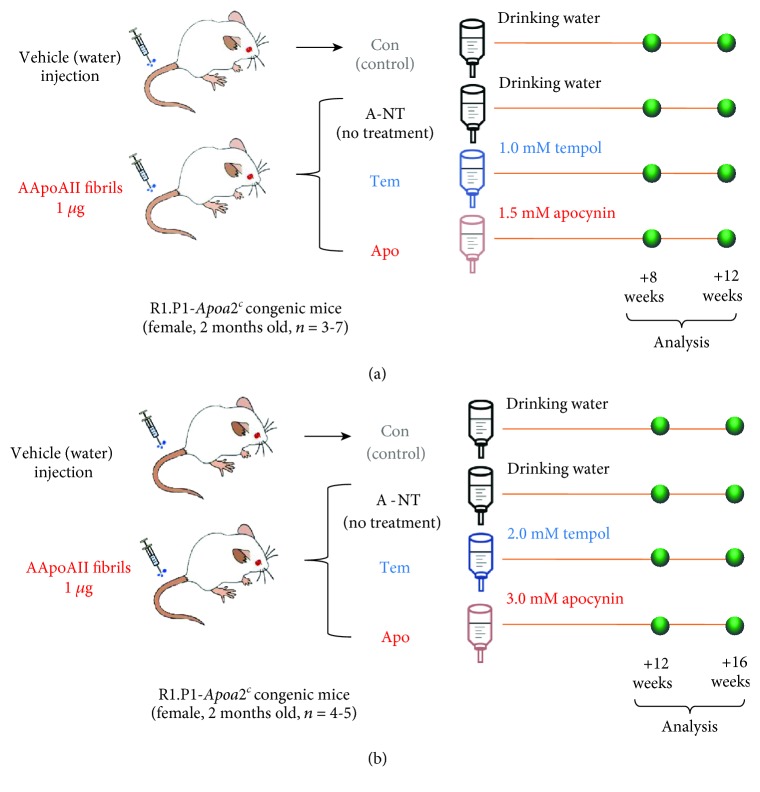
Experimental design. (a) Two-month-old female R1.P1-*Apoa2c* mice were divided into 4 groups: control (Con), no treatment (A-NT), tempol (Tem), and apocynin (Apo). AApoAII amyloidosis was induced in the mice in the A-NT, Tem, and Apo groups by injection with 1 *μ*g AApoAII fibrils in the tail vein; the Con group was injected with distilled water instead of AApoAII fibrils. Concomitantly, the Tem and Apo group mice were treated with either tempol (1 mM) or apocynin (1.5 mM) in drinking water for 8 or 12 weeks (*N* = 3–7 per group). (b) In the high-dose series, the experiment was repeated with double doses of tempol (2 mM) or apocynin (3 mM) and the experimental period was 12 and 16 weeks (*N* = 4 or 5 per group).

Two-month-old female R1.P1-*Apoa2^c^* mice were divided into 4 groups: control (Con), no treatment (A-NT), tempol (Tem), and apocynin (Apo). AApoAII amyloidosis was induced in mice in the A-NT, Tem, and Apo groups by injection with 1 *μ*g AApoAII fibrils in the tail vein; the Con group was injected with distilled water (DW) instead of AApoAII fibrils (Figure 1(a)). Concomitantly, the Tem and Apo group mice were treated with either the free radical scavenger tempol (1 mM) or the NADPH oxidase inhibitor apocynin (1.5 mM) in drinking water for 8 or 12 weeks (*N* = 3-7 mice per group). For the high-dose experimental series, R1.P1-*Apoa2^c^* mice were treated with 2 mM tempol or 3 mM apocynin in drinking water for 12 or 16 weeks (*N* = 4 or 5 mice per group) (Figure 1(b)). Doses of apocynin and tempol were chosen in accordance with previous reports [34–36]. The drug solution was prepared prior to use. Tempol (Cat No. 176141) and apocynin (Cat No. w508454) were purchased from Sigma-Aldrich (Tokyo, Japan).

#### 2.2.2. Induction of AApoAII Amyloidosis

We isolated AApoAII amyloid fibrils using Pras' method [37] from the liver of a 14-month-old R1.P1-*Apoa2^c^* mouse having heavy amyloid deposits and injected 1 *μ*g amyloid fibrils in each mouse to induce AApoAII amyloidosis followed the protocols of Li et al. [33]. Before injection, the fibril samples were sonicated (Fig.  S1) and used immediately as described previously [20].

### 2.3. Histology

#### 2.3.1. Amyloid Fibril Detection

Amyloid deposits were detected as apple-green birefringence in organ sections stained with a saturated solution of 1% Congo red dye under polarizing light microscopy (LM) (Axioskop 2, Carl Zeiss, Tokyo, Japan). An amyloid score (from 0 to 4) in each organ was determined semiquantitatively as described previously [21]. Two observers having no information of the tissue evaluated the grade of amyloid deposition in Congo red stained tissue. The degree of amyloid deposition in each mouse was represented by an amyloid index (AI) which is the average of the amyloid scores in seven organs (heart, liver, spleen, stomach, small intestine, tongue, and skin) [21, 33, 38].

#### 2.3.2. Immunohistochemistry

AApoAII and AA fibrils were also detected by immunohistochemistry (IHC) with specific rabbit antiserum against mouse ApoA-II or mouse AA, which were produced against guanidine hydrochloride-denatured AApoAII or AA fibrils in our laboratory [20, 21, 33, 38]. Four-micron (4 *μ*m) thick sections of fixed organs were treated with 3% H_2_O_2_ in methanol for 30 minutes (min) to inactivate endogenous peroxidase and were blocked with 5% bovine serum albumin in PBS. The sections were incubated overnight at 4°C with rabbit antisera against mouse ApoA-II (1 : 3000) and AA (1 : 3000) prepared in our laboratory [39] or 4-hydroxynonenal (4-HNE) (1 : 300, Abcam plc, Cambridge, UK), followed by incubation with the biotinylated secondary antibody (Abcam plc). Target proteins were identified by the horseradish peroxidase-labeled streptavidin-biotin method (DAKO, Glostrup, Denmark). In a negative control section, the first antibody was omitted to confirm the specificity of staining. To analyze the positive area in each organ quantitatively, the ratios of the positively stained area to a whole organ in randomly captured 5 areas under ×200 or ×400 magnification were measured using image processing program (NIH ImageJ software, version 1.61) [33].

#### 2.3.3. Semiquantification of Oxidative Stress

Two blinded observers, who had no information regarding the tissue, observed 4-HNE stain intensity in tissue specimens of IHC. We evaluated the stain intensity in every organ. The stain intensity was scored as follows: 0 (absent), 1 (few), 2 (mild), 3 (middle), and 4 (severe). The average of these scores by two observers represented the final 4-HNE evaluations for statistical analyses by the Steel-Dwass test.

### 2.4. Biochemical Analysis

#### 2.4.1. Malondialdehyde (MDA)

Lipid peroxidation was analyzed using a Lipid Peroxidation Colorimetric/Fluorometric Assay Kit (BioVision, San Francisco CA, USA) by means of malondialdehyde (MDA) content in accordance with the instructions provided by the manufacturer. The 10 mg liver stored at -80°C was homogenized to detect the MDA concentration followed by the methods as described previously [40]. Three technical replicates were conducted for each sample. The MDA content is calculated by comparing the measured values to a calibration curve prepared using an MDA standard (BioVision). The coefficient of variation (*r* ^2^) for the calibration curve was 0.99.

#### 2.4.2. High-Density Lipoprotein (HDL)

We determined the HDL-cholesterol levels in the plasma using quantitative assay kits (HDL-cholesterol E test, Wako, Osaka, Japan) [33, 38, 41].

### 2.5. Immunoblot Analysis

We followed the methods as described previously [33, 42, 43] to determine the level of plasma apolipoproteins. Plasma samples were separated on Tris-Tricine/SDS–16.5% polyacrylamide gels electrophoresis (PAGE) as follows: 0.5 *μ*L plasma for ApoA-I or ApoE and 1 *μ*L plasma for ApoA-II. After electrophoresis, proteins were transferred to a polyvinylidene difluoride membrane (Immobilon, 0.2 *μ*m pore, Millipore Corp., MA, USA). The membrane was incubated with primary antibody solution, polyclonal rabbit anti-mouse ApoA-I antiserum produced in our laboratory [41, 42] (diluted 1 : 4000), or the ApoA-II antiserum (diluted 1 : 3000) or ApoE antibody (1 : 500, Santa Cruz, San Francisco, CA, USA) for 1 hour at room temperature and then overnight at 4°C. Next, the horseradish peroxidase-conjugated anti-rabbit IgG (Code #7074, Cell Signaling Technology Inc., Danvers MA, USA) (1 : 3000) was used for 1-hour incubation at room temperature. ApoA-I, ApoA-II, and ApoE were detected with the enhanced chemiluminescence (ECL) method, and the target protein levels were analyzed using the NIH ImageJ software.

### 2.6. Gene Expression Analysis

We followed a previously described method to analyze the mRNA expression [33, 44]. Quantitative real-time PCR analysis was carried out using an ABI PRISM 7500 Sequence Detection system (Applied Biosystems) with SYBR Green (TaKaRa Bio, Tokyo, Japan). The *β*-actin gene was used to normalize gene expression. The forward and reverse primer sequences for real-time PCR are listed in Table S1. Chemical reagents in the experiments, unless otherwise specified, were obtained from Wako Pure Chemical Industries Ltd. (Osaka, Japan).

### 2.7. Statistical Analyses

All data are presented as the mean ± S.D. For the comparison of the parametrical data, one-way analysis of variance (ANOVA) with key's test was performed using the SPSS 25.0 software package (Abacus Concepts, Berkley, CA USA). For the comparison of the nonparametrical data, the Kruskal-Wallis test with the Steel-Dwass test was performed. *p* values less than 0.05 were considered to be statistically significant.

## 3. Results

### 3.1. AApoAII Amyloidosis Significantly Declined in the 12-Week Intake Groups, Especially in the Stomach, Skin, and Liver

To examine whether oxidative stress contributes to the process of AApoAII amyloidosis, R1.P1-*Apoa2^c^* mice were administered the ROS scavenger tempol (1 mM) or the NADPH oxidase inhibitor apocynin (1.5 mM) in drinking water for 8 or 12 weeks beginning at 8 weeks of age. At 7 weeks old, we measured the body weight, food intake, and water consumption of the mice and monitored abnormal conditions of the mice for one week. At 8 weeks old, we divided the mice into 4 groups and confirmed that these data were not different among the 4 groups and that no obvious abnormal performance was observed in any of the mice. The Con group, which had no induction of AApoAII amyloidosis, nor drug intervention, was used as a control group to provide baseline data. The A-NT, Tem, and Apo groups were injected with 1 *μ*g of AApoAII amyloid fibrils through the tail vein at 8 weeks of age to induce AApoAII amyloidosis. Concomitantly, the Tem and Apo groups were administered the respective drugs in drinking water (Figure 1(a)). There was no difference in body weight, food intake, and water consumption among the 4 groups during the experiments (Fig.  S2). To observe the effect of drug dose on the experimental results, a high-dose (twice the initial dose) experiment was repeated, and the time points were determined at 12 weeks and 16 weeks (Figure 1(b)).

**Figure 2:**
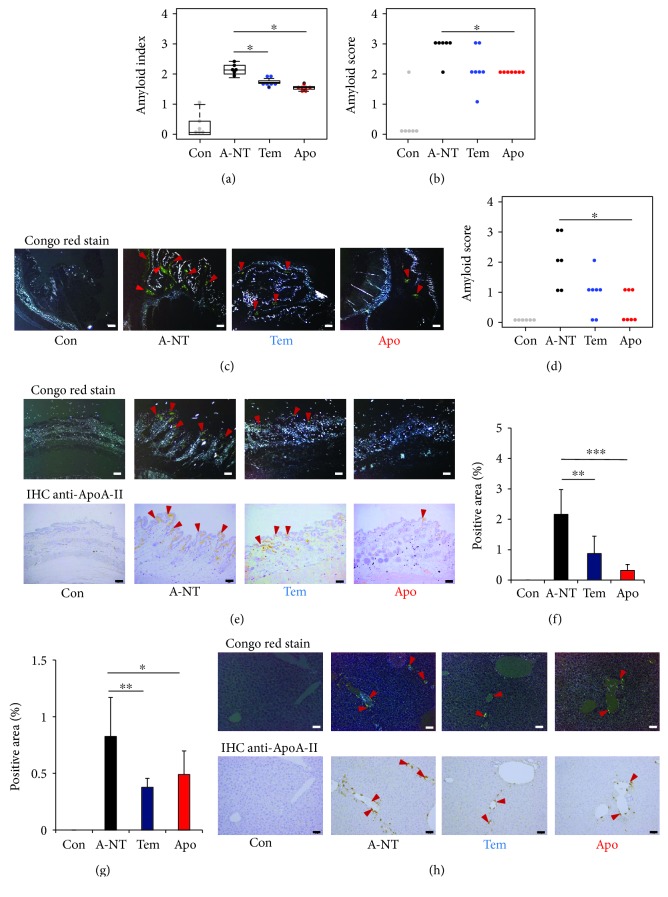
AApoAII amyloidosis significantly declined in the 12-week-intake groups. (a) Amyloid index (AI) of the 12-week groups. (b) Amyloid score of the 12-week groups in the stomach. (c) Representative LM images of AApoAII deposits in the stomach in induced mice. Amyloid deposits (red arrows) were identified by green birefringence in Congo red-stained sections using polarized LM. Each scale bar indicates 100 *μ*m. (d) Amyloid score of the 12-week groups in the skin. (e) Representative LM and IHC images of AApoAII deposits in the skin in induced mice. AApoAII deposits were confirmed by IHC with anti-ApoA-II antiserum. Amyloid deposits are indicated by red arrows. Each scale bar indicates 100 *μ*m. Comparisons of positive areas of amyloid deposits in the (f) skin and (g) liver. (h) Representative LM and IHC images of AApoAII deposits in the liver in induced mice. Amyloid deposits are indicated by red arrows. Each scale bar indicates 50 *μ*m. *N* = 6 (Con and A-NT) and 7 (Tem and Apo). Results are shown as box and whisker plots, where a box extends from the 25th to the 75th percentile with the median shown as a line in the middle and whiskers indicate the smallest and largest values (a). Each dot represents an individual mouse (a, b, d). Each symbol and bar represent the mean ± S.D. (f, g). ^∗^ *p* < 0.05, ^∗∗^ *p* < 0.01, and ^∗∗∗^ *p* < 0.001, the Kruskal-Wallis test with the Steel-Dwass test for the amyloid index and amyloid score (a, b, d). The Tukey-Kramer method for the multiple comparison of the IHC-positive area (f, g).

After 8 weeks, we collected several organs (heart, liver, spleen, stomach, small intestine, tongue, skin, lung, and kidney) for the evaluation of amyloid deposits. First, the amyloid deposits were graded by the presence of apple-green color birefringence in Congo red-stained tissue under polarized LM. Small green birefringence signals were observed in the heart, small intestine, tongue, stomach, and lung, but not in the liver, spleen, skin, or kidney (Fig.  S3a). However, the degree of amyloid deposition was not significantly different among the 3 induced groups (Fig.  S3b). At 12 weeks postinjection, amyloid protein deposition was found in all seven organs, except the spleen (Fig.  S4), and the amyloid indices (AIs) were significantly lower in the Tem and Apo groups than in the A-NT group (Figure 2(a)). We then analyzed the degree of amyloid deposition in each organ and found that the effect of oxidative stress inhibitors was inconsistent among the different organs and that significant inhibitory effects appeared in the stomach and skin compared with the A-NT group (Figures 2(b)–2(f)), with apocynin exhibiting a stronger inhibitory effect than tempol. IHC was used to confirm the type of amyloid protein with anti-ApoA-II and anti-AA antiserum (Figure 2(e) and Fig.  S5) and for quantitative analysis of AApoAII amyloid load in the skin using an image processing program (Figure 2(f)). Results showed a significant suppression of AApoAII amyloid deposition in the skin following 12-week supplementation with oxidative stress inhibitors. Although the amyloid scores of the livers in the 3 induced groups were nearly identical, we found that the oxidative stress inhibitors showed significant inhibitory effects in amyloid deposition in the liver by calculating the ApoA-II positive area (Figures 2(g) and 2(h)).

### 3.2. Decrease in Oxidative Stress Levels following Supplementation with Oxidative Stress Inhibitors

**Figure 3:**
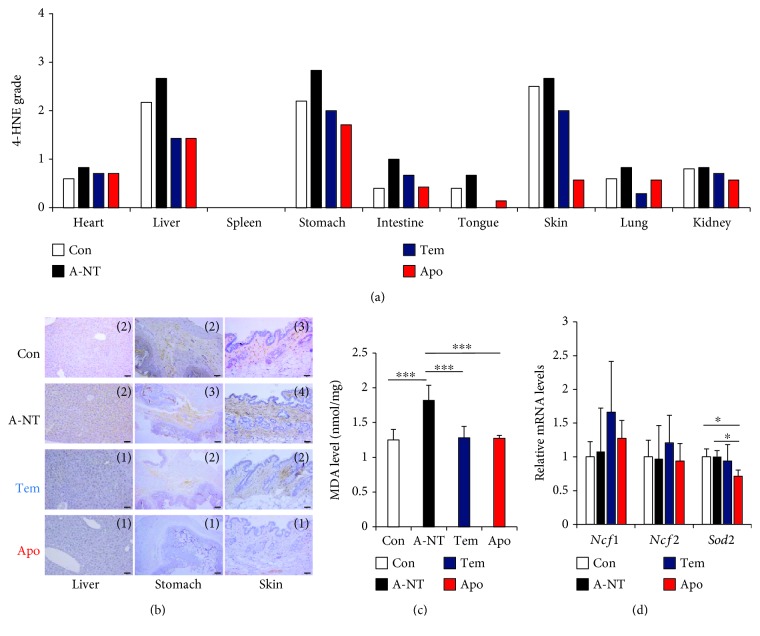
Detection of oxidative stress levels following daily supplementation with oxidative stress inhibitors for 12 weeks. (a) The schema of grades of 4-HNE in every organ following IHC detection. Each column represents the mean of the grade. (b) Representative images of 4-HNE IHC of the liver, stomach, and skin. The grades of each section are shown on the upper-right of the image. Each scale bar indicates 50 *μ*m. (c) MDA levels in the liver. (d) mRNA expression levels of genes related to oxidative stress response in the liver. Histograms show the fold change in mRNA levels relative to the Con group. Each column and bar represent the mean ± S.D. *N* = 6 (Con and A-NT) and 7 (Tem and Apo). ^∗^ *p* < 0.05 and ^∗∗∗^ *p* < 0.001, the Tukey-Kramer method for multiple comparison.

To verify whether tempol or apocynin suppressed oxidative stress levels *in vivo*, we detected lipid peroxidation marker 4-HNE in various organs using IHC. The results showed that 4-HNE was slightly increased in the A-NT group compared with the Con group and that intake of oxidative stress inhibitors reduced oxidative stress in the liver, stomach, and skin, in which higher basal values of 4-HNE were observed (Figures 3(a) and 3(b)). Based on the grading of the staining intensity, the effects of oxidative stress inhibitors showed a trend towards a decline in the liver and a significant decline in the skin (Fig.  S6). Moreover, we found that another oxidative stress marker in the liver, namely, MDA, increased in the A-NT group, but not in the Tem and Apo groups. These results suggest that oxidative stress levels may have increased more in the A-NT group than in the Con group in the liver, stomach, and skin and that this change was inhibited upon intake of oxidative stress inhibitors (Figure 3(c)). To confirm the rise in oxidative stress at the gene level, we also determined expression levels of the oxidative stress-related genes *Ncf1* and *Ncf2*, which code for the subunit of NADPH oxidase [45] and *SOD2*, respectively, in the liver.  Figure 3(d) shows that *Sod2* expression was suppressed by supplementation with apocynin but that there was no change in *Ncf1* and *Ncf2*.

### 3.3. Effects on Plasma ApoA-II Protein and HDL Concentrations and Liver *Apoa2* mRNA Expression following Intake of Oxidative Stress Inhibitors

**Figure 4:**
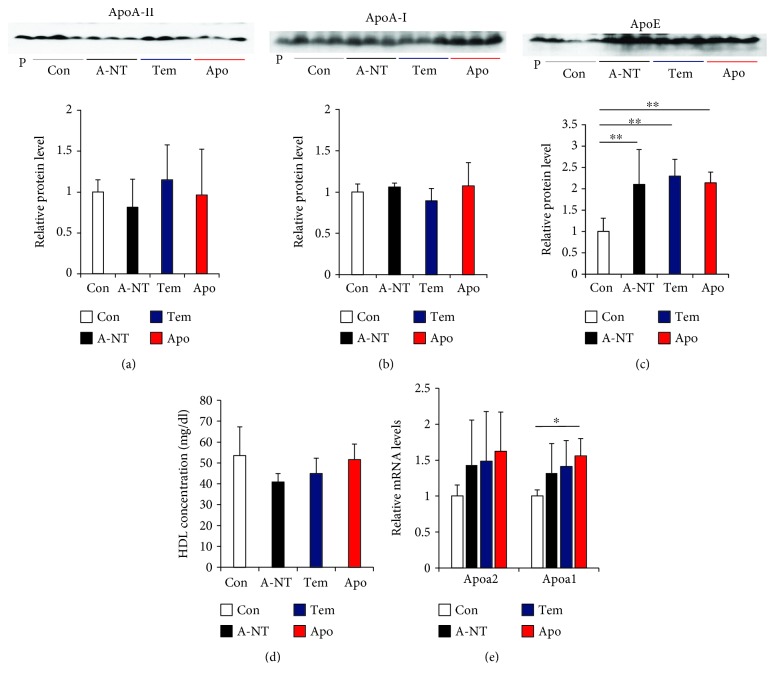
Effects of daily supplementation with oxidative stress inhibitors on plasma lipoproteins. (a–c) Concentrations of ApoA-II, ApoA-I, and ApoE proteins in the plasma were determined by densitometry of Western blot after SDS-PAGE of the plasma. The representative results of Western blot are shown above the figures. There was no difference in ApoA-II or ApoA-I plasma concentrations; however, the concentration of ApoE increased significantly in the 3 amyloidosis-induced groups. Histograms show fold changes relative to the Con group and represent the means ± S.D. P indicates, the pooled plasmas of female R1.P1-Apoa2c mice at 2 months of age (*N* = 4) that did not have AApoAII amyloid deposits, as the positive control of these proteins. (d) HDL plasma concentration was determined using quantitative assay kits. (e) mRNA expression levels of ApoA-II and ApoA-I in the liver were determined by quantitative real-time PCR and shown by fold changes relative to the Con group. *N* = 6 (Con and A-NT) and 7 (Tem and Apo). ^∗^ *p* < 0.05 and ^∗∗^ *p* < 0.01, the Tukey-Kramer method for multiple comparison.

To investigate whether oxidative stress inhibitors directly decrease plasma concentrations of precursor ApoA-II protein or HDL and result in suppression of AApoAII amyloid deposition, we used immunoblotting of the plasma to detect levels of ApoA-II, ApoA-I, and ApoE (Figures 4(a)–4(c)). Results showed that tempol and apocynin had no effect on plasma concentrations of ApoA-II and ApoA-I. However, we found that ApoE levels increased significantly in the induced groups. Moreover, plasma HDL levels were similar across groups (Figure 4(d)). As both ApoA-I and ApoA-II are produced in the liver, we also detected the expression of *Apoa2* and *Apoa1* mRNA levels in the liver by real-time PCR and found that *Apoa1* expression was upregulated in the induced group (Apo) compared with the Con group, but no difference was observed between the induced groups. The *Apoa2* gene did not differ significantly among all groups (Figure 4(e)).

### 3.4. Antioxidative Treatment Downregulates Gene Expression of ER Stress in the Liver and May Improve the Microenvironment

**Figure 5:**
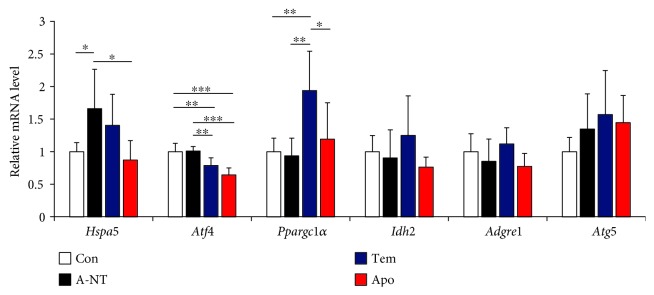
Determination of the effects of antioxidative treatment on mRNA expression levels of the genes related to UPR. Using real-time RT-PCR, we determined mRNA expression levels of the following genes in the liver: UPR sensor gene (*Hspa5* and *Atf4*), mitochondrial function-related gene (*Ppargc1α* and *Idh2*), macrophage marker gene (*Adgre1*), and autophagy-related gene (*Atg5*). Each column represents fold changes relative to the Con group and the mean ± S.D. *N* = 6 (Con and A-NT) and 7 (Tem and Apo). ^∗^ *p* < 0.05, ^∗∗^ *p* < 0.01, and ^∗∗∗^ *p* < 0.001, the Tukey-Kramer method for multiple comparison.

To further investigate the effects of oxidative stress inhibitors on the microenvironment of tissues, we determined the mRNA levels of genes involved in ER stress (*Hspa5* and *Atf4*) in the liver (Figure 5). The mRNA levels of *Hspa5* were increased in the A-NT group, but were suppressed in the Apo group; levels of *Atf4* were significantly decreased in the Tem and Apo groups compared with the Con and A-NT groups. We also determined the expression levels of the mitochondrial function-related factors *Ppargc1α* and *Idh2*, macrophage marker *Adgre1* (F4/80), and autophagy-related factor *Atg5.* The levels of these genes were not different among any of the groups, except for upregulated levels of *Ppargc1a* in the Tem group.

### 3.5. The High-Dose Series Exhibited a Consistent Preventive Effect on Amyloid Deposition

**Figure 6:**
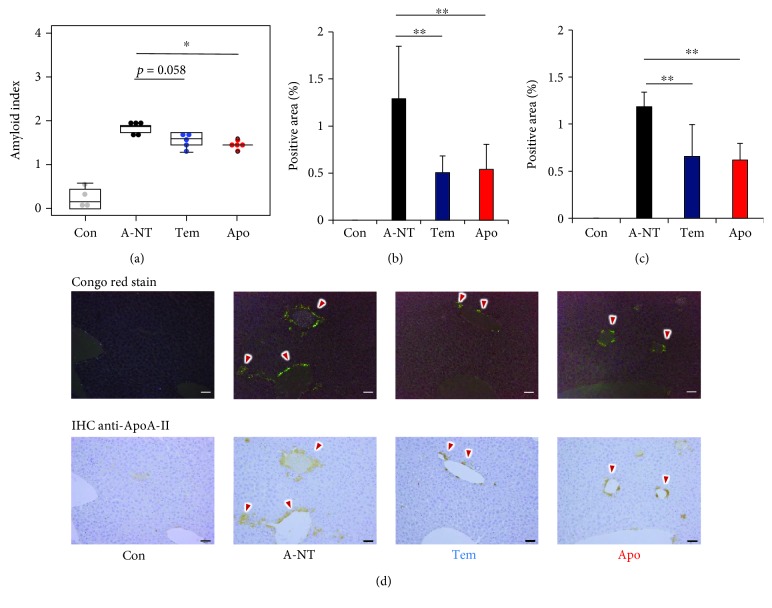
High-dose supplementation exhibits a consistent preventive effect against amyloidosis with the initial dose. (a) The amyloid index was significantly lower in the Tem and Apo groups compared with the A-NT group after 12-week-supplementation. Comparison of IHC-positive areas showed decreased amyloid deposition in the Tem and Apo groups in the (b) skin and (c) liver. (d) Representative polarized LM and IHC images of AApoAII deposits (red arrows) in the liver in induced mice. Results are shown as box and whisker plots, where a box extends from the 25th to the 75th percentile with the median shown as a line in the middle and whiskers indicate the smallest and largest values. Each dot represents an individual mouse (a). Each scale bar indicates 50 *μ*m (d). Each column and bar represents the mean ± S.D. (b, c) *N* = 4 (Con) and 5 (A-NT, Tem, and Apo). ^∗^ *p* < 0.05, the Kruskal-Wallis test with the Steel-Dwass test (a). ^∗∗^ *p* < 0.01, the Tukey-Kramer method for multiple comparison (b, c).

To determine the effect of different drug doses of oxidative stress inhibitors on amyloidosis progression, the drug dose was doubled and the experiment was repeated with modified incubation times (i.e., 12 and 16 weeks after induction of amyloidosis). Consistent with the initial-dose series, a significant suppression of amyloid deposition also occurred after 12-week intake of oxidative stress inhibitors, based on both AI and skin amyloid scores (Figure 6(a) and Fig.  S7a). ApoA-II IHC was subsequently performed, and decreased levels were observed in the liver and skin of treated animals compared to the A-NT group, in both the 12-week and 16-week groups (Figures 6(b)–6(d) and Fig.  S8b-d). However, following the increase in the degree of deposition, we did not observe significant decreases in AI among the induced groups after 16-week intake (Fig.  S7b and Fig.  S8a).

## 4. Discussion

### 4.1. Oxidative Stress Is Involved in AApoAII Amyloid Formation

With aging, the balance between production and removal of ROS is altered and levels of oxidative stress gradually increase to cause oxidative damage to lipids, proteins, and DNA, leading to cellular dysfunction and various age-related diseases [46, 47]. Numerous studies have investigated the relationship between oxidative stress and pathogenesis of amyloidosis, especially on localized brain amyloidosis and amyloid-related neurodegenerative diseases: Alzheimer's disease, cerebral amyloid angiopathy (CAA), amyotrophic lateral sclerosis (ALS), etc. [8, 11, 13, 16, 34, 36, 48–50]. However, a clear understanding of the specific mechanism responsible for the acceleration of amyloidosis and neurodegeneration caused by oxidative stress remains elusive. Unfortunately, there are still insufficient reports supporting the contribution of oxidative stress to the pathogenesis of systemic amyloidosis and related adverse effects on tissues [51].

In this study, supplementation with oxidative stress inhibitors for 12 weeks led to decreases in mouse AApoAII amyloidosis and demonstrated that inhibition of oxidative stress was effective at suppressing systemic amyloid deposition *in vivo*. Meanwhile, our results suggest that the effect of antioxidants varied depending on the specific organs. In the amyloid-induced groups, amyloid scores in the stomach and skin were significantly decreased by supplementation with tempol and apocynin, with the preventive effect being most distinct in the skin (Figure 2). Although the amyloid scores evaluating the grading of amyloid deposition in the liver were not significantly different between groups with and without oxidative stress inhibitors, quantitatively measuring the positive area of ApoA-II deposition in IHC demonstrated a significant preventive effect of oxidative stress inhibitors against amyloid deposition in the liver. We did not observe this change in other organs, including the heart, spleen, small intestine, tongue, lung, and kidney.

Our recent study found that following caloric restriction, the degree of amyloid deposition in almost all organs (except for the stomach) was significantly decreased [33]. These results suggest that caloric restriction may prevent amyloid deposition, not only via the same pathway as that of oxidative stress inhibitors but also via other mechanisms as well. The two oxidative stress inhibitors investigated in the present study exhibited different degrees of inhibition. Apocynin showed a relatively stronger preventive effect against amyloid deposition in the stomach and skin than tempol (Figures 2(a), 2(b), 2(d), and 2(f)), although the preventive effects of the two inhibitors were nearly identical in the liver (Figure 2(g)). By measuring the degree of oxidative stress using 4-HNE and MDA as markers, we identified relatively high baseline levels of oxidative stress in the liver, stomach, and skin and found that apocynin reduced oxidative stress more strongly in the stomach and skin compared with tempol (Figure 3(a)), but to similar degrees in the liver (Figure 3(c)). These differences may be due to differences in the main sources of oxidative stress within each organ [46, 47, 52–54]. We considered that these organs are susceptible to induction of oxidative stress by external influences depending on their function [52–54] and that relatively high levels of baseline oxidative stress may be related to the antiamyloidogenic effects of the antioxidants. In a study of CAA, antioxidants were found to reduce amyloid deposition, improve vascular conditions, and reduce the occurrence of complications, but the same changes did not appear in the amyloid deposits of the brain parenchyma [36]. Further experiments are needed to confirm our hypothesis that the effect of antioxidant treatments on amyloidosis depends on the oxidative stress state of the target organs and may provide support for the use of antioxidant treatments in the clinic.

We repeated the second experiment with a higher antioxidant dose to confirm the results of the first experiment and to determine dose effects on the inhibition of amyloidosis. Because amyloid deposits were not been observed in the liver and skin in the 8-week groups of the initial dose experiment, we chose a longer incubation time and sacrificed mice at 12 weeks and 16 weeks after induction of amyloidosis in the second high-dose series. Results were similar to those of the initial-dose experiment and suggest that 1.0 mM tempol and 1.5 mM apocynin are sufficient to show significant preventive effects.

### 4.2. Oxidative Stress May Suppress Amyloid Deposition by Affecting Protein Stability

Considering that various factors affect AApoAII amyloid deposition, we investigated whether oxidative stress inhibitors directly affect levels of precursor ApoA-II protein and HDL. Results showed that neither levels of *Apoa2* mRNA expression in the liver nor plasma levels of ApoA-II protein were significantly changed by supplementation with oxidative stress inhibitors. In our previous study, we showed that one possible mechanism of suppression of amyloid deposition by caloric reduction is a reduction in plasma concentrations of ApoA-II and ApoA-II/ApoA-I ratios [33]. These findings suggest that oxidative stress inhibitors suppress AApoAII amyloid deposition, not by reducing plasma concentrations or synthesis of ApoA-II, but by a pathway distinct from that of caloric reduction.

We considered the second possibility for reducing amyloid deposition by increasing amyloid protein stability. During the pathogenesis of amyloidosis, oxidative modification of amyloid proteins may play a key role. Oxidative conditions could modify the proteins and alter the structure to one with a higher aggregation propensity [8–10, 55–57]. Several experiments confirmed that antioxidants help to maintain protein stability and can reduce amyloid formation *in vivo* [36, 51, 58]. Low pH states cause protein instability and promote amyloid fibril formation of A*β*, TTR, and ApoA-II *in vitro* [29, 38, 59]. Results of these previous studies suggest that increased levels of oxidative stress may induce a change in the local microenvironment to an acidic pH [60]. Reports by Saito et al. have shown that tissues exhibiting TTR amyloid deposition were under oxidative stress and that both the Val30Met mutant and wild-type TTR proteins had higher ratios of S-nitrosylation, with the ability to form amyloid fibrils *in vitro* [9]. Polyphenolic compounds such as curcumin are known to exhibit antioxidative effects and prevent fibril formation by binding to amyloid proteins [61–63]. Thus, preventing the destabilization of amyloid proteins may offer a target of oxidative stress inhibitors, such as tempol and apocynin. Future experiments allowing for the accurate measurement of the oxidation state of AApoAII amyloid protein *in vivo* should provide greater insight into the relationship between amyloidosis and oxidative stress.

### 4.3. Decreased Oxidative Stress May Improve the Microenvironment Involved in Amyloid Fibril Formation

Real-time PCR results suggest a significant decline in the degree of UPR and ER stress in the liver by supplementation with oxidative stress inhibitors (Figure 5). Our previous studies have shown that ER stress in the liver and kidney increased with the appearance and progression of AApoAII amyloidosis [28]. Thus, the decreased amyloid deposition caused by oxidative stress inhibitors may reduce *Hspa5* expression. Expression levels of *Atf4*, a transcription factor involved in various intracellular stress signaling pathways (e.g., ER stress and mitochondrial stress [64, 65]), were decreased by oxidative stress inhibitors. Mitochondrial dysfunction caused by amyloid deposition is considered to be the most important cause of subsequent cell dysfunction and death. In our previous report, we demonstrated that caloric reduction improved mitochondrial function and reduced AApoAII amyloid deposition [33]. However, in the present study, the expression of *Ppargc1α and Idh2* did not change with amyloid deposition and the presence of oxidative stress inhibitors. Moreover, macrophage (*Adgre1*) and autophagy (*Atg5*) markers also did not change with amyloid deposition and antioxidant treatment. Further studies are needed to clarify the relevant molecular mechanisms by which treatment with antioxidants can improve the microenvironment involved in amyloid fibril deposition.

### 4.4. Plasma ApoE Is Increased in the Early Stages of ApoA-II Amyloidosis

ApoE is a chaperone protein that is widely found to be associated with various amyloid depositions and is considered a risk factor for the onset of amyloidosis [66–68]. In Alzheimer's disease, different isoforms of ApoE play different roles in the pathogenesis of amyloidosis: ApoE4 has been shown to promote or inhibit the assembly of A*β* protein into filaments in both *in vitro* and *in vivo* mouse models [69, 70]. Our recent studies have shown that AApoAII amyloid fractions isolated from the liver exhibited a large number of codeposited amyloid-associated proteins, which may contribute to the progression of amyloid deposition [71]. Plasma concentrations of ApoE, which is the most abundant amyloid-associated protein, were gradually decreased and associated with an increasing degree of amyloid deposition [71]. Interestingly, plasma concentrations of ApoE significantly increased in the amyloidosis-induced groups compared with the Con group in the present study. These results suggest that amyloid deposition may stimulate the upregulation of ApoE protein in the early stages of amyloidosis and provide new evidence that ApoE is closely involved in the pathogenesis of amyloidosis. Supplementation with antioxidants did not have an effect on increasing ApoE plasma levels.

## 5. Conclusion

Taken together, our results provide evidence that oxidative stress is involved in the progression of amyloidosis. The formation and deposition of amyloid fibrils are the result of a combination of various factors. Although the application of oxidative stress inhibitors has a certain inhibitory effect on amyloid deposition, it is not enough to completely block the progression of amyloidosis. Results of the present study suggest that antioxidants mainly reduce the levels of oxidative stress, can help to maintain protein stability, and may improve the cellular microenvironment. Oxidative stress inhibitors offer a therapeutic strategy that should be considered for the future treatment of amyloidosis.

## Data Availability

The data used to support the findings of this study are included within the article.

## Supplementary Materials

Supplementary Materials Figure S1: detection of AApoAII amyloid fibrils for injection. Abundant AApoAII amyloid fibrils were observed in the injected solution. Scale bar is 50 nm. Figure S2: body weight, food intake, and water consumption were measured weekly. (a) Body weight of 8-week groups and 12-week groups. Each column and bar represent the mean ± S.D. (*N* = 3-7). (b) Food intake and (c) water consumption of each cage were measured weekly. Data are represented in g/mouse/d (food intake) and mL/mouse/d (water consumption). Figure S3: no significant differences were observed in amyloid deposition among the three induced groups in the 8-week groups. (a) Amyloid scores of the 8-week groups in each organ. Each column represents the mean. (b) Amyloid indices of the 8-week groups. Amyloid indices were not significantly different among the three induced groups. The amyloid index (AI) was calculated as the average of the amyloid scores in seven organs (heart, liver, spleen, tongue, stomach, small intestine, and skin). Results are shown as box and whisker plots, where a box extends from the 25th to the 75th percentile with the median shown as a line in the middle and whiskers indicate the smallest and largest values. Each dot represents an individual mouse. (c) 4-HNE grade in each organ. Each column represents the mean. *N* = 3. The Kruskal-Wallis test with the Steel-Dwass test for the amyloid index and amyloid score. Figure S4: amyloid scores of the 12-week groups in each organ. The amyloid index (AI) was calculated as the average of the amyloid scores in seven organs (heart, liver, spleen, tongue, stomach, small intestine, and skin). Each column represents the mean. *N* = 6 or 7. Figure S5: no AA amyloid deposition was detected in this experiment. Representative IHCs with anti-ApoA-II or AA antisera in the intestine, liver, and tongue of amyloidosis-induced mice. No detectable AA staining was observed in any organ. Each scale bar indicates 100 *μ*m. Figure S6: statistical analysis of 4-HNE grade in 12-week groups. (a) 4-HNE grade in the liver, (b) stomach, and (c) skin. Results are shown as box and whisker plots, where a box extends from the 25th to the 75th percentile with the median shown as a line in the middle and whiskers indicate the smallest and largest values. Each dot represents an individual mouse. ^∗^ *p* < 0.05 vs. Con, the Kruskal-Wallis test with the Steel-Dwass test for 4-HNE grade. *N* = 6 (Con and A-NT) or 7 (Tem and Apo). Figure S7: amyloid scores of high-dose 12-week and 16-week groups in each organ. The amyloid index (AI) was calculated as the average of the amyloid scores in seven organs (heart, liver, spleen, tongue, stomach, small intestine, and skin). Each column represents the mean. *N* = 4 or 5. Figure S8: the high-dose oxidative stress inhibitors exhibited the same level of an antiamyloid effect on R1.P1-*Apoa2^c^* mice for 16 weeks. (a) Amyloid indices of the high-dose 16-week groups. Comparison of positive areas of amyloid deposition in the skin (b) and liver (c). (d) Representative IHC images of AApoAII deposits in the liver of induced mice. Results are shown as box and whisker plots, where a box extends from the 25th to the 75th percentile with the median shown as a line in the middle and whiskers indicate the smallest and largest values. Each dot represents an individual mouse (a). Each column and bar represent the mean ± S.D. (b, c). Each scale bar indicates 100 *μ*m. *N* = 4 (Con, A-NT, and Tem) and 5 (Apo). ^∗^ *p* < 0.05, ^∗∗^ *p* < 0.01, and ^∗∗∗^ *p* < 0.001, the Tukey-Kramer method for multiple comparison of an IHC-positive area. Table S1: the specific primers for real-time PCR.
