# Supplementary material for: Suppression of Mouse AApoAII Amyloidosis Progression by Daily Supplementation with Oxidative Stress Inhibitors
Source: Oxid Med Cell Longev. 2019 Jun 4;2019:1263274. doi: 10.1155/2019/1263274 (PMC6589291; doi:10.1155/2019/1263274)
Supplement: Supplementary Materials — Figure S1: detection of AApoAII amyloid fibrils for injection. Abundant AApoAII amyloid fibrils were observed in the injected solution. Scale bar is 50 nm. Figure S2: body weight, food intake, and water consumption were measured weekly. (a) Body weight of 8-week groups and 12-week groups. Each column and bar represent the mean ± S.D. (N = 3-7). (b) Food intake and (c) water consumption of each cage were measured weekly. Data are represented in g/mouse/d (food intake) and mL/mouse/d (water consumption). Figure S3: no significant differences were observed in amyloid deposition among the three induced groups in the 8-week groups. (a) Amyloid scores of the 8-week groups in each organ. Each column represents the mean. (b) Amyloid indices of the 8-week groups. Amyloid indices were not significantly different among the three induced groups. The amyloid index (AI) was calculated as the average of the amyloid scores in seven organs (heart, liver, spleen, tongue, stomach, small intestine, and skin). Results are shown as box and whisker plots, where a box extends from the 25th to the 75th percentile with the median shown as a line in the middle and whiskers indicate the smallest and largest values. Each dot represents an individual mouse. (c) 4-HNE grade in each organ. Each column represents the mean. N = 3. The Kruskal-Wallis test with the Steel-Dwass test for the amyloid index and amyloid score. Figure S4: amyloid scores of the 12-week groups in each organ. The amyloid index (AI) was calculated as the average of the amyloid scores in seven organs (heart, liver, spleen, tongue, stomach, small intestine, and skin). Each column represents the mean. N = 6 or 7. Figure S5: no AA amyloid deposition was detected in this experiment. Representative IHCs with anti-ApoA-II or AA antisera in the intestine, liver, and tongue of amyloidosis-induced mice. No detectable AA staining was observed in any organ. Each scale bar indicates 100 μm. Figure S6: statistical analysis of 4-HNE grade in [file 1263274.f1.pdf]

Supplemental Data

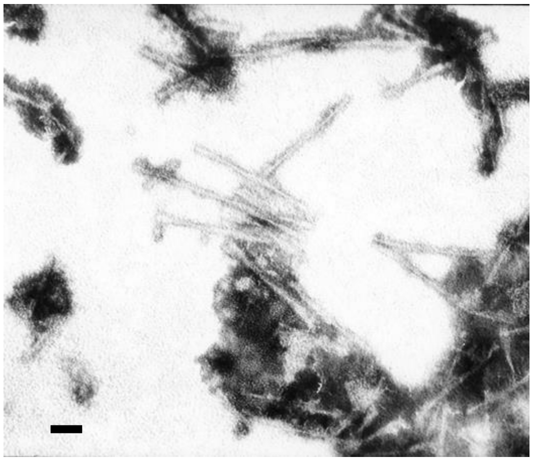

Figure S1. Detection of AApoAII amyloid fibrils for injection. Abundant AApoA-II amyloid fibrils were observed in the injected solution. Scale bar is 50 nm.

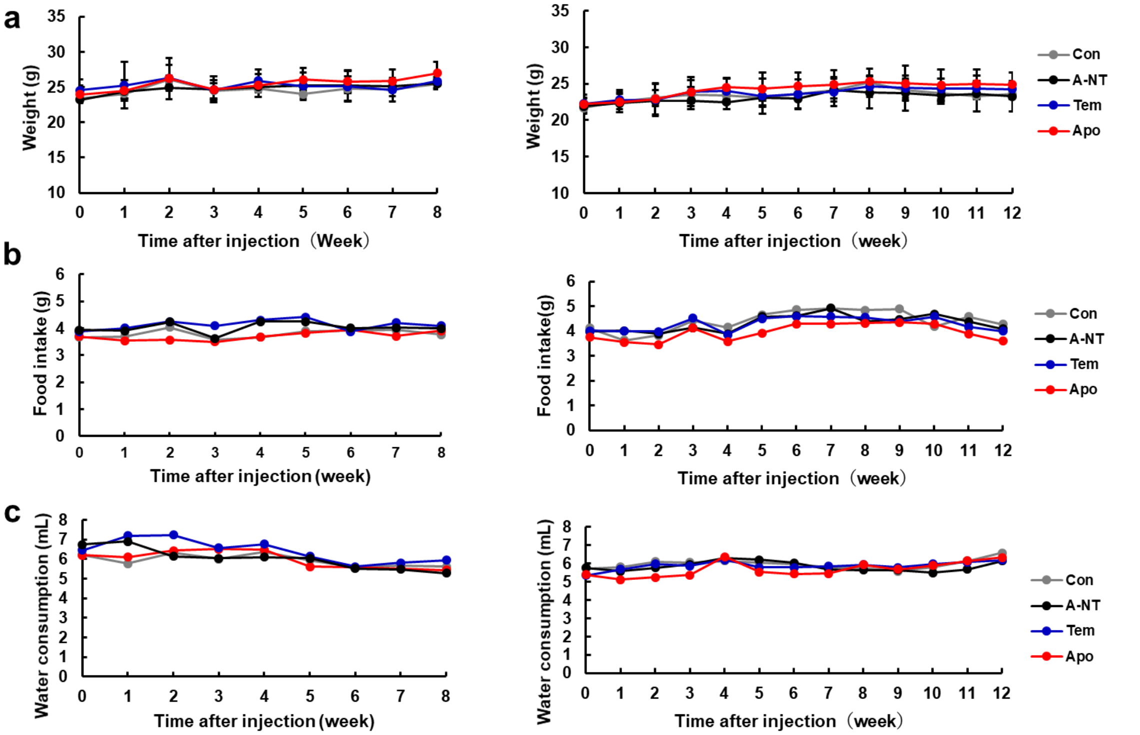

Figure S2. Body weight, food intake and water consumption were measured weekly. (a) Body weight of 8-week groups and 12-week groups. Each column and bar represent the mean ± S.D. (N = 3-7). (b) Food intake and (c) water consumption of each cage were measured weekly. Data are represented in g/mouse, day (food intake) and mL/mouse, day (water consumption).

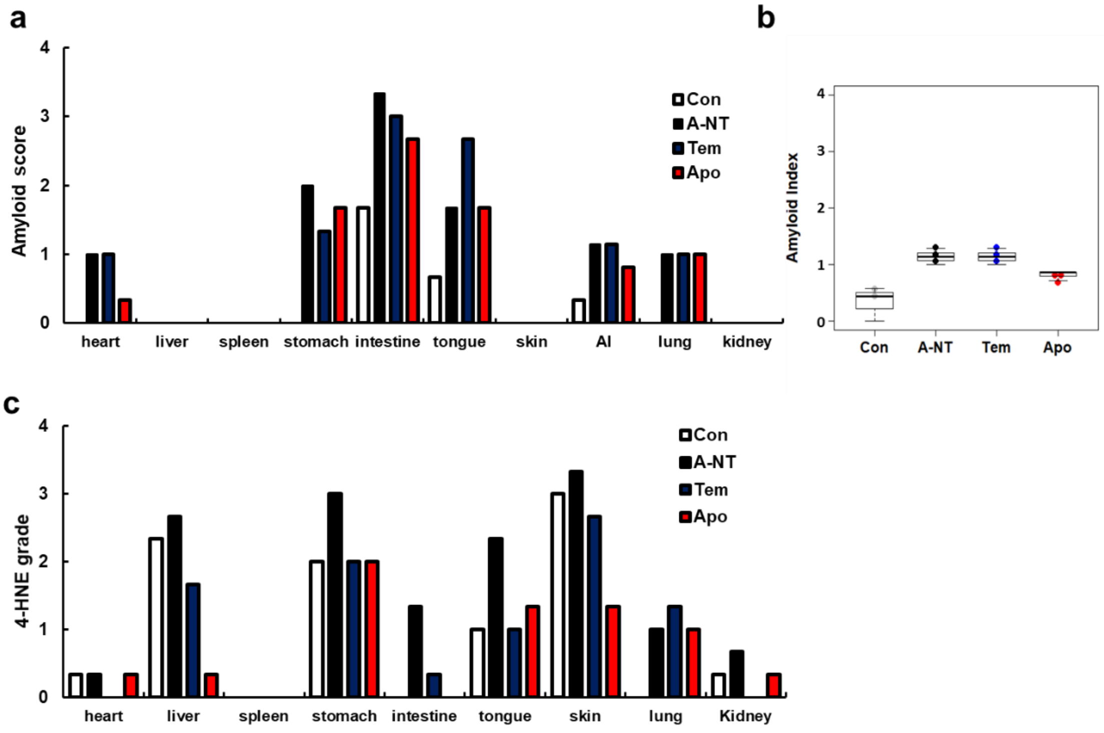

Figure S3. No significant differences were observed in amyloid deposition among the three induced groups in the 8-week groups. (a) Amyloid scores of the 8-week groups in each organ. Each column represents the mean. (b) Amyloid indices of the 8-week groups. Amyloid indices were not significantly different among the three induced groups. The amyloid index (AI) was calculated as the average of the amyloid scores in seven organs (heart, liver, spleen, tongue, stomach, small intestine, and skin). Results are shown as box-and-whisker plots, where a box extends from the 25th to the 75th percentile with the median shown as a line in the middle, and whiskers indicate the smallest and largest values. Each dot represents an individual mouse. (c) 4-HNE grade in each organ. Each column represents the mean. N = 3. Kruskal-Wallis test with Steel-Dwass test for amyloid index and amyloid score.

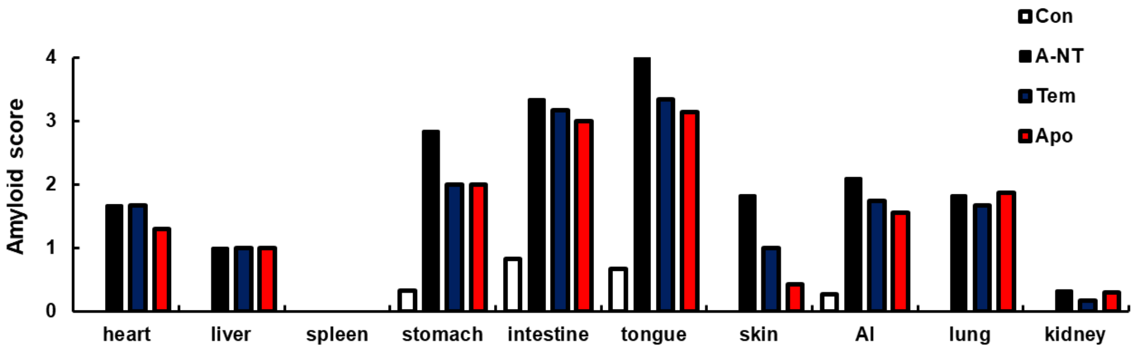

Figure S4. Amyloid scores of the 12-week groups in each organ. The amyloid index (AI) was calculated as the average of the amyloid scores in seven organs (heart, liver, spleen, tongue, stomach, small intestine, and skin). Each column represents the mean. N = 6 or 7.

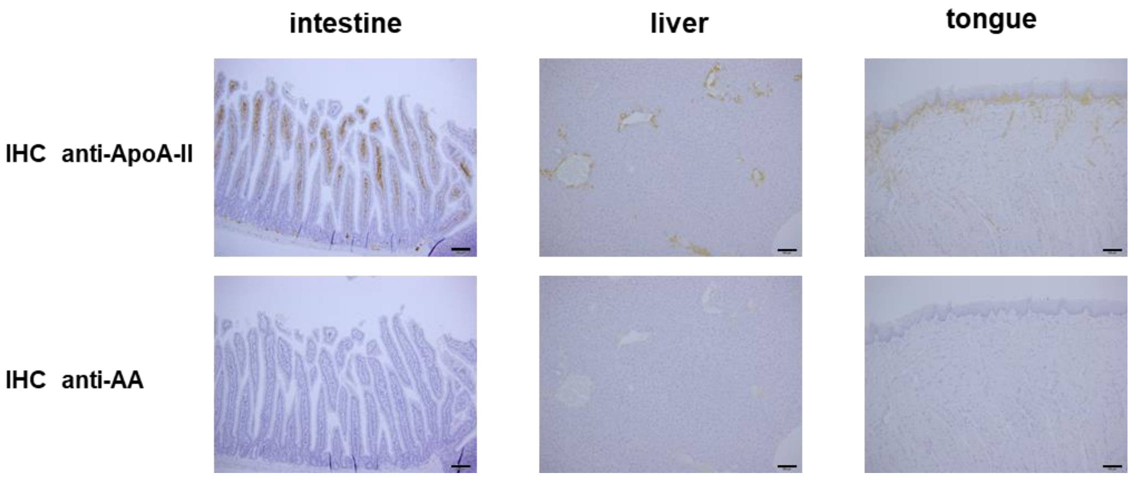

Figure S5. No AA amyloid deposition was detected in this experiment. Representative IHCs with anti-ApoA-II or AA antisera in the intestine, liver and tongue of amyloidosis-induced mice. No detectable AA staining was observed in any organ. Each scale bar indicates 100 μm.

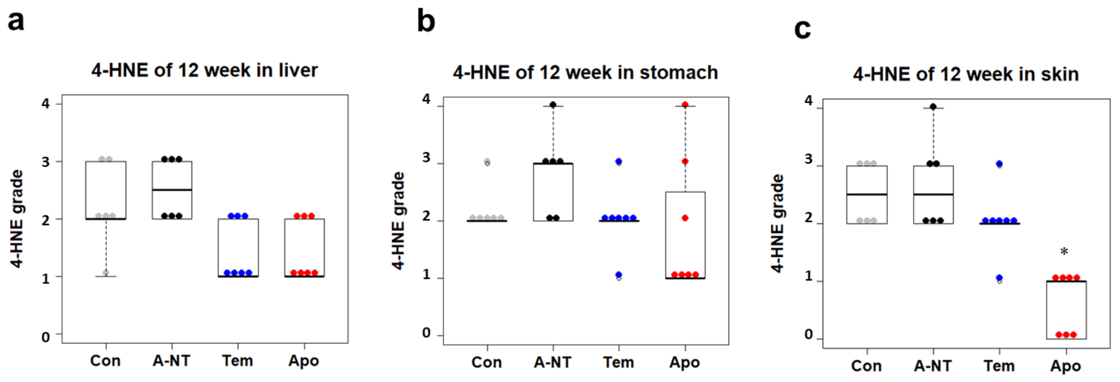

Figure S6. Statistical analysis of 4-HNE grade in 12-week groups. (a) 4-HNE grade in the liver, (b) stomach and (c) skin. Results are shown as box-and-whisker plots, where a box extends from the 25th to the 75th percentile with the median shown as a line in the middle, and whiskers indicate the smallest and largest values. Each dot represents an individual mouse. * $p<0.05$ vs Con, Kruskal-Wallis test with Steel-Dwass test for 4-HNE grade. N = 6 (Con and A-NT) or 7 (Tem and Apo).

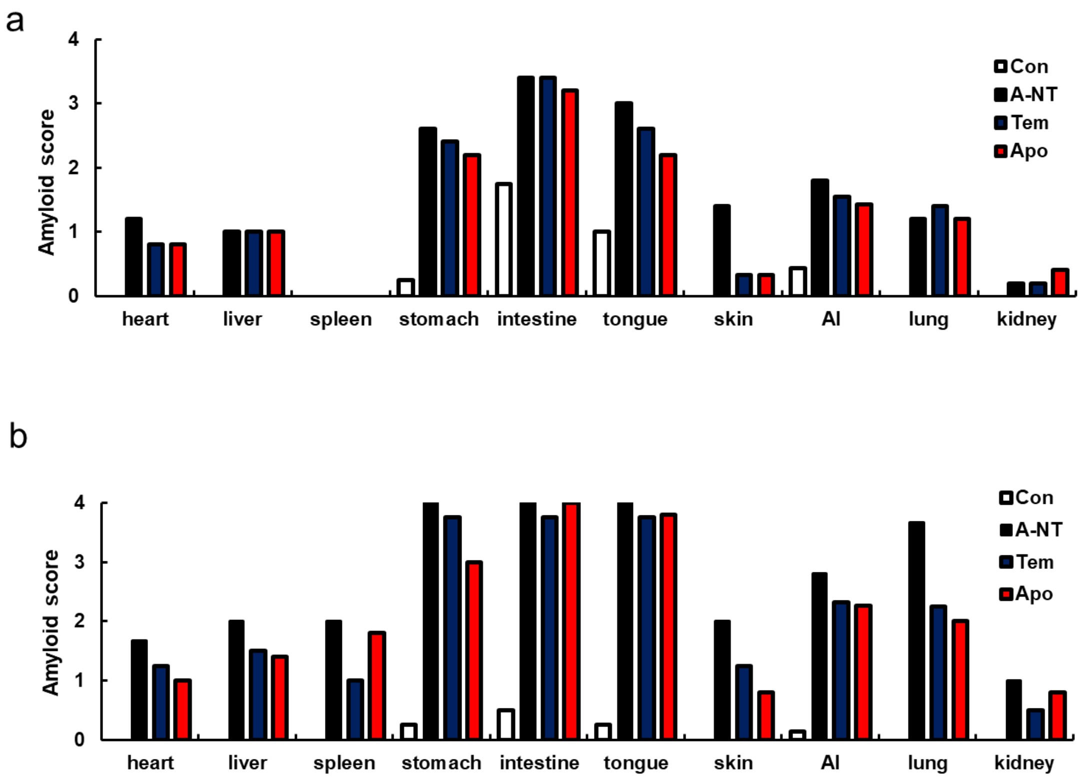

Figure S7. Amyloid scores of high-dose 12-week and 16-week groups in each organ. The amyloid index (AI)

was calculated as the average of the amyloid scores in seven organs (heart, liver, spleen, tongue, stomach, small intestine, and skin). Each column represents the mean. N = 4 or 5.

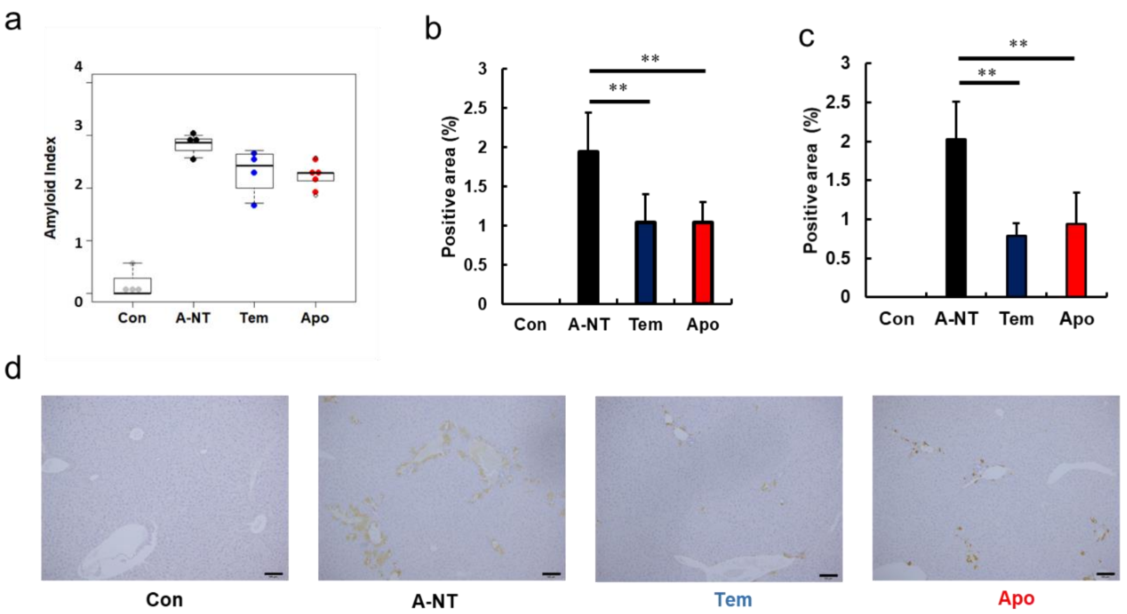

Figure S8. The high-dose oxidative stress inhibitors exhibited the same level of anti-amyloid effect on R1.P1-*Apoa2*^{c} mice for 16 weeks. (a) Amyloid indices of the high-dose 16-week groups. Comparison of positive areas of amyloid deposition in the skin (b) and liver (c). (d) Representative IHC images of AApoAII deposits in the liver in induced mice. Results are shown as box-and-whisker plots, where a box extends from the 25th to the 75th percentile with the median shown as a line in the middle, and whiskers indicate the smallest and largest values. Each dot represents an individual mouse (a). Each column and bar represent the mean ± S.D. (b and c). Each scale bar indicates 100 µm. N = 4 (Con, A-NT and Tem) and 5 (Apo). *p<0.05, **p<0.01, *** p<0.001, Tukey-Kramer method for multiple comparison of IHC positive area.

Table S1. The specific primers for real-time PCR.

Apoa1	F: GTGGCTCTGGTCTTCCTGAC
	R: ACGGTTGAACCCAGAGTGTC
Apoa2	F: GCCTGTTCACTCAGTACTTTCAG
	R: CAGACTAGTTCCTGCTGACC
Adgre1	F: GATGAATTCCCGTGTTGTTGGT
	R: ACATCAGTGTTCCAGGAGACACA
Ncf1	F: ATCCTATCTGGAGCCCCTGA
	R: CACCTGCGTAGTTGGGATCC
Ncf2	F: CAGACCCAAAACCCCAGAAA
	R: AAAGCCAAACAATACGCGGT
Atg5	F: AGAGTCAGCTATTTGACGTTGG
	R: TGGACAGTGTAGAAGGTCCTTTT
Hspa5	F: ACCCCGAGAACACGGTCTT
	R: TGCCCACCTCCAATATCAACT
Ppargc1α	F: AAGTGTGGAACTCTCTGGAACTG
	R: GGGTTATCTTGGTTGGCTTTATG
β-Actin	F: ACAATGAGCTGCGTGTGGCC
	R: CCTCGTAGATGGGCACAGTG
Idh2	F: GGCTGTCAAGTGTGCCACAATC
	R: TTGGCTCTCTGAAGACGGTTCC
Sod2	F: CCGAGGAGAAGTACCACGAG
	R: GCTTGATAGCCTCCAGCAAC
Atf4	F: CCTTCGACCAGTCGGGTTTG
	R: CTGTCCCGGAAAAGGCATCC
